# Enhancing Fracture Resistance by Customizing Glass Fiber Posts in Endodontically Treated Teeth: Protocol for a Systematic Review

**DOI:** 10.2196/76027

**Published:** 2026-02-17

**Authors:** Sanika Damle, Manoj Chandak, Unnati Shirbhate, Pavan Bajaj

**Affiliations:** 1 Department of Conservative Dentistry and Endodontics, Sharad Pawar Dental College, Datta Meghe Institute of Higher Education and Research, Sawangi (Meghe), Wardha, Maharashtra, 442001, India, +91 7447401822; 2 Department of Periodontics and Oral Implantology, Sharad Pawar Dental College, Datta Meghe Institute of Higher Education and Research, Wardha, Maharashtra, India

**Keywords:** root canal, fracture resistance, cemented posts, custom made fiber posts, endodontic therapy

## Abstract

**Background:**

The loss of tooth structure in endodontically treated teeth compromises their structural integrity and increases their vulnerability to fractures. To strengthen these teeth, post and core systems must be used. Among post materials, glass fiber has become more and more common because of it advantageous mechanical and aesthetic qualities. However, the effectiveness of customized glass fiber posts in enhancing fracture resistance compared to prefabricated posts remains a subject of ongoing debate.

**Objective:**

This systematic review primarily focuses on the fracture resistance of endodontically treated teeth reinforced with customized glass fiber posts. It aims to assess how well they function in terms of resilience, flexibility, and failure patterns in comparison to prefabricated fiber posts and other post materials.

**Methods:**

To locate relevant studies published up to 2025, a comprehensive literature search will be conducted through various materials available electronically on websites such as Google Scholar, Web of Science, PubMed, Semantic Scholar, and Scopus. The selection criteria will include randomized controlled clinical trials that evaluate fracture resistance of customized glass fiber posts in teeth that have had endodontic treatment. The research will be screened using predetermined standards for inclusion and exclusion. Quality assessment and collection of data will be performed strictly, in accordance with Preferred Reporting Items for Systematic Reviews and Meta-Analysis (PRISMA) guidelines. To examine the results quantitatively, a meta-analysis will be conducted, if at all possible, with a random effects model for heterogeneity. Statistical methods will be used for evaluating the effectiveness of custom glass fiber posts, alternative post materials, and prefabricated posts.

**Results:**

By comparing the mean differences in fracture resistance of teeth treated endodontically using various post systems, the effectiveness of customized glass fiber posts will be assessed. Other aspects to be examined include general biomechanical performance, failure mechanisms, and stress distribution. This study will use a random effects model to estimate the combined effect size measurements and the corresponding 95% CIs. We anticipate that the data synthesis will be conducted between February and March 2026 for this systematic review and will be finished by April or July 2026.

**Conclusions:**

Customized glass fiber posts may show promise in strengthening teeth that have undergone endodontic treatment by lowering the risk of failure and increasing fracture resistance. Even if there is current evidence that they are more effective than prefabricated posts, we need to synthesize the quality of the evidence. Therefore, this review aims to evaluate whether customized glass fiber posts effectively enhance fracture resistance in endodontically treated teeth.

## Introduction

### Rationale

Endodontics, one of the major fields in dentistry, focuses on the causes, prevention, diagnosis, and management of conditions and traumas affecting the dental pulp and periapical tissues [1]. Today, the emphasis of dental therapy has switched to a more conservative approach, although in the past, extraction was the recommended treatment for the majority of teeth that were highly carious. When the vital contents of the canal are removed by endodontic therapy, the tooth becomes pulpless and has calcified tissues with much lower moisture content than living teeth [2]. Because of these alterations, the tooth becomes weaker and more prone to fracture. To restore the strength and integrity of such a tooth, post and core systems are commonly used [3].

Typically, a post is used to increase core retention, which increases crown retention [4]. Over the past 10 years, the most frequently used post types in dentistry have been cast posts and cores. These typically need an extra laboratory step where the impressions taken from the prepared post area are used to create a custom post [3]. Despite the widespread use of cast or prefabricated metallic posts, there are certain issues that have been identified with these systems, such as corrosion, root fractures, loss of retention, the need to remove considerable root structure, and concentration of stress [5].

Fiber-reinforced composite (FRC) posts are a popular option for already endodontically treated teeth. A milestone was reached with the introduction of carbon fiber–reinforced resin posts, and this changed some of the underlying concepts used to restore previously endodontically treated teeth. Glass fiber posts are now most often used to improve the distribution of masticatory stresses and guarantee greater retention and support for restorative material, leading to better recovery of severely injured teeth. Prefabricated posts and specially customized chair-side posts are the two main types of FRC posts [6].

Prefabricated FRC posts are made by inserting a great volume of continuous, 1-directional, reinforcing fibers into a polymer matrix. Usually composed of materials like carbon, glass, or quartz fibers, these posts require little preparation before being placed. Conversely, non-preimpregnated fibers, usually made of glass or polyethylene, are shaped into posts during the restoration process to make chair-side customized FRC posts [7]. To treat teeth severely affected by cavities or trauma, custom-made posts and cores are sometimes advised. Because they fit precisely in the prepared post space, they are especially helpful for teeth with curved or elliptical canals [8].

### Review Question

Is there any role for customized glass fiber posts in fracture resistance for endodontically treated teeth?

### Objective

We aimed to evaluate the fracture resistance, mechanical stability, and long-term durability of customized glass fiber posts in endodontically treated teeth and to compare their effectiveness with traditional post systems.

## Methods

### Eligibility Criteria

Studies will be selected based on predefined inclusion and exclusion criteria to ensure consistency and reproducibility in the review process (Textbox 1).

Textbox 1. **Inclusion and exclusion criteria**

**Inclusion criteria**

- Study design: randomized controlled trials, controlled clinical trials, and in vitro experimental studies evaluating fracture resistance
- Participants: patients with endodontically treated teeth
- Intervention: customized glass fiber posts for postendodontic restoration
- Comparison: any conventional post system or no intervention
- Outcome: fracture resistance of the restored teeth
- Language: publications in English

**Exclusion criteria**

- Study design: case reports, reviews, editorials, conference abstracts, and letters to the editor
- Participants: studies involving teeth that were not endodontically treated

### Information Sources

To find papers that meet the review’s eligibility criteria, electronically available databases will be searched, including Web of Science, PubMed, Cochrane Library, Google Scholar, Semantic Scholar, and Scopus.

### Search Strategy

To locate relevant papers, a limited search of MEDLINE will be conducted. Using terms from the abstracts, titles, and keywords, a thorough search strategy will be created. The following search strategy will be followed: “endodontically treated teeth”[Title/Abstract] OR “endodontically treated tooth”[Title/Abstract] OR “root canal treated teeth”[Title/Abstract] OR “root canal treated tooth”[Title/Abstract] OR “endodontic treatment*”[Title/Abstract] OR “RCT”[Title/Abstract] OR “root canal treatment*”[Title/Abstract] OR “root canal therap*”[Title/Abstract] OR “endodontic therap*”[Title/Abstract] OR “root canal therapy”[MeSH Terms] AND “customized glass fiber post*”[Title/Abstract] OR “custom made fiber post*”[Title/Abstract] OR “custom-made fiber post*”[Title/Abstract] OR “glass fiber post*”[Title/Abstract] OR “glass fiber post*”[Title/Abstract] OR “endodontic post*”[Title/Abstract].

### Study Selection, Data Management, and Data Collection Process

The three reviewers (SD, US, PB) will consult the eligibility criteria when deciding which research papers to include. Recording the selection will help with duplicate removal and record management. Studies will be screened blindly, and disagreements will be resolved through discussion. After loading the retrieved studies, 2 researchers will screen the abstracts and titles. A full-text review screening will be conducted by 2 authors, and any disagreements will be solved by a fourth reviewer (MC).

A data extraction tool used on the included papers will serve as a guide for the reviewers. The randomized controlled trials will include individuals who are in good systemic health as well as those undergoing endodontic treatment. Pregnant women and those with a history of systemic sickness will be excluded. The findings obtained will be narratively summarized. Methodological, statistical, and clinical heterogeneity will be examined and evaluated. If all of the incorporated studies and their findings are found to be sufficiently homogeneous, a quantitative synthesis, also known as a meta-analysis, will be conducted.

### Data Items

The extracted data will be duplicated for each of the 2 blinded reviewers (US and SD). A Microsoft Excel spreadsheet will be used to store the extracted data, which will contain publication information, the setting of the research study, the fiber post, push-out bond strength, and luting cement. Customized glass fiber posts, fracture resistance, and endodontic clinical parameters will all be considered as clinical determinants.

### Outcomes and Prioritization

The primary outcome is the fracture resistance of teeth that have previously received endodontic treatment and restoration with customized glass fiber posts, measured by fracture load or strength. Secondary outcomes include the failure mode (adhesive, cohesive, or mixed failure), the strength of the bond between the post and dentin, and long-term durability under functional stress. These outcomes will help assess the mechanical stability and clinical efficacy of customized posts compared to conventional systems.

### Risk of Bias

To assess the methodological quality and risk of bias of the included studies, standardized and validated tools will be use. For randomized controlled trials, the Risk of Bias 2 (RoB 2) tool, developed by the Cochrane Collaboration, will be used. This tool evaluates five domains: (1) bias arising from the randomization process, (2) bias due to deviations from intended interventions, (3) bias due to missing outcome data, (4) bias in measurement of the outcome, and (5) bias in selection of the reported results. Each domain will be rated as “low risk,” “some concerns,” or “high risk,” and an overall risk of bias judgment will be made accordingly.

If any in vitro studies are included in the review, they will be assessed using the QUIN (Quality Assessment Tool for In Vitro Studies), which considers methodological rigor, sample size justification, standardization of procedures, and statistical analysis, among other relevant criteria.

The risk of bias assessments will be independently conducted by 2 reviewers (US and SD). In case of disagreement, third and fourth reviewers (PB and MC) will act as arbiters to reach consensus. To minimize bias, all reviewers will remain blinded to each other’s assessments during the evaluation process.

### Data Synthesis and Assessment of Heterogeneity

A random effects model will be used to account for potential heterogeneity across studies. Statistical heterogeneity will be assessed using the *I*^2^ statistic and the chi-square test. If the studies are deemed sufficiently homogeneous in terms of design, population, and outcome measures, a quantitative synthesis (meta-analysis) will be performed.

Effect sizes will be reported as standardized mean differences for continuous outcomes (eg, fracture resistance values) with 95% CIs. If binary outcomes are present, odds ratios will be calculated.

All analyses will be conducted using appropriate statistical software, such as RevMan (Review Manager; version 5.4) or Stata (version 17.0, StataCorp LLC). This approach adheres to the guidelines outlined in the *Cochrane Handbook for Systematic Reviews of Interventions* [9] to ensure methodological transparency and reproducibility.

## Results

**Figure 1.**
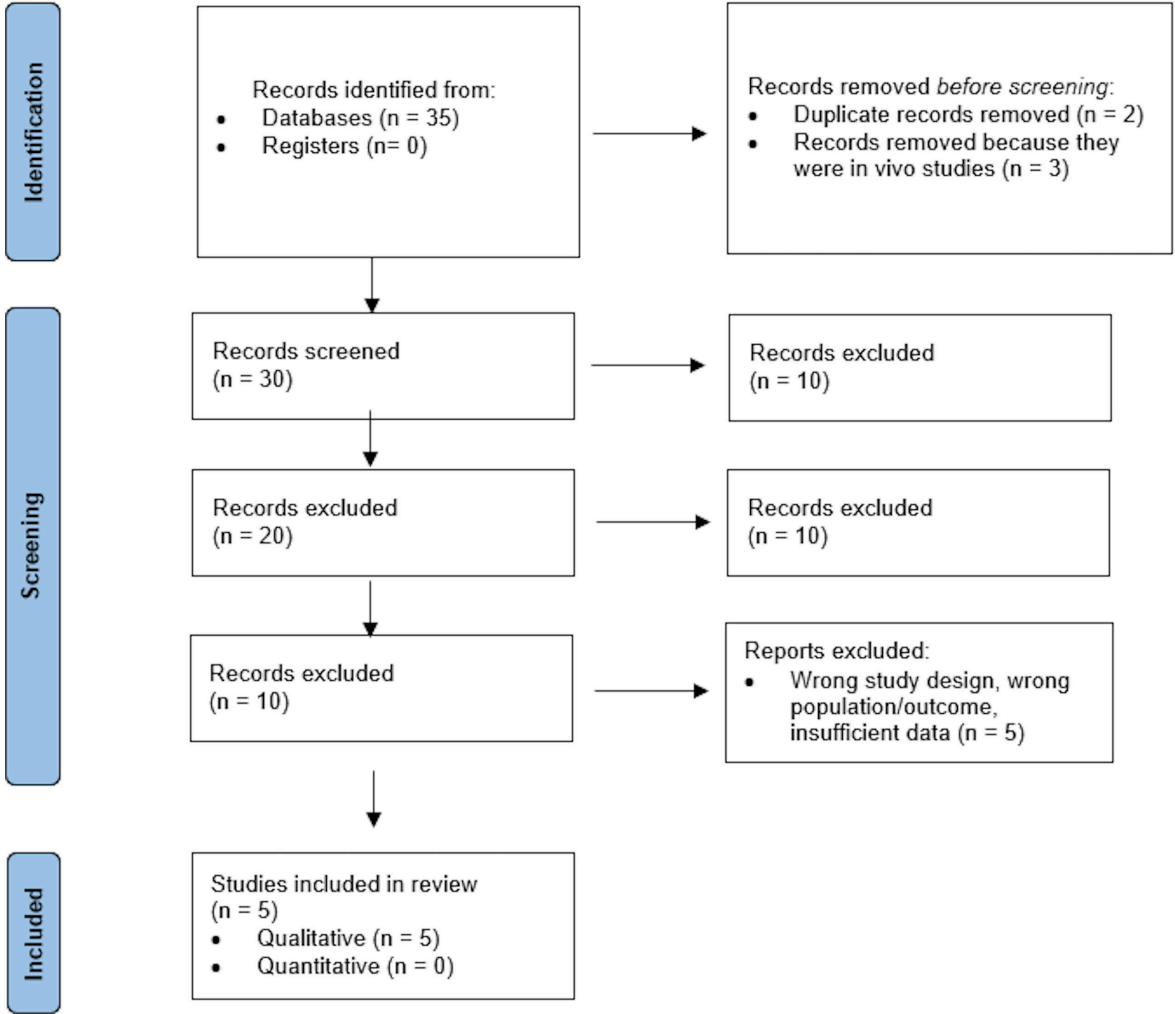
PRISMA (Preferred Reporting Items for Systematic Review and Meta-Analysis) flowchart.

Identification, screening, and inclusion of studies is expected to be completed in May of 2026. At every stage of the process, the 2 reviewers were blinded. Team meetings were conducted on a regular basis to settle disputes and resolve conflicts between reviewers, increasing the transparency of the evaluation procedure. Every discussion was documented and recorded. When the systematic review is complete, it will be submitted for publication in accordance with the recommendations in the Preferred Reporting Items for Systematic Review and Meta-Analysis Protocols (PRISMA-P) checklist. Figure 1 shows the PRISMA flowchart for this review.

As the literature search and screening processes were completed in May 2025, preliminary data on the number of studies identified, screened, and included have been documented and are presented in the PRISMA flowchart in Figure 1. These numbers are subject to change as full-text screening and risk of bias assessment progress.

## Discussion

### Anticipated Findings

This systematic review will assess the effectiveness of customized glass fiber posts for fracture resistance in endodontically treated teeth. Because of their improved stress distribution and increased adaptation to the root canal shape, we anticipate that customized glass fiber posts will offer better fracture resistance in comparison with prefabricated ones [10] due to greater retention and less stress concentration, resulting from the customized posts’ superior fit to the canal walls. Customized posts enable a more conservative approach, maintaining the tooth’s structural integrity, in contrast to prefabricated fiber posts, which frequently necessitate significant dentin removal to fit the canal [11].

The intrinsic properties of glass fiber posts, such as their elasticity modulus, which is the same as dentin, contribute to improved stress distribution and a reduced incidence of catastrophic root fractures. Customization further enhances these properties by eliminating gaps between the post and canal walls, minimizing microleakage and improving the overall bonding interface with the adhesive cement [5]. We anticipate that the findings of this review will show the importance of post customization in achieving optimal fracture resistance in endodontically treated teeth. Clinicians should consider factors such as canal morphology, residual dentin thickness, and adhesive bonding techniques when selecting and customizing posts. The use of CAD/CAM technology or direct fiber post buildup techniques can further enhance the fit and mechanical performance of customized posts [7].

While the current evidence supports the superiority of customized glass fiber posts, some limitations should be acknowledged. The heterogeneity of study designs, sample sizes, and testing methodologies may impact the generalizability of the results [12]. Additionally, a sufficient number of clinical studies are required for assessing the outcomes of customized posts in functional conditions. Customized glass fiber posts offer significant advantages over prefabricated posts in terms of fracture resistance, biomechanical compatibility, and adhesive bonding. Their ability to preserve dentin and enhance stress distribution makes them a favorable choice for restoring endodontically treated teeth. Further clinical investigations are required to establish standardized protocols for their fabrication and long-term effectiveness [13,14].

### Strengths and Limitations

Strengths of this systematic review include the clinical relevance of an evaluation of customized glass fiber posts for enhancing fracture resistance and mechanical stability in endodontically treated teeth, as well as comprehensive outcome measures, such as mechanical performance, fracture patterns, fracture resistance, and failure modes. The use of multiple high-quality databases and standardized tools for risk of bias assessment (RoB 2 and QUIN) to improve methodological rigor is also a strength.

Limitations include the inclusion of in vitro studies, which may not accurately replicate clinical conditions; potential heterogeneity in study design, materials, and testing protocols across included studies; and the limited availability of long-term clinical data. Additionally, while gray literature sources like Google Scholar will be used (with caution), there remains a risk of publication bias and variable study quality.

### Conclusion and Directions for Dissemination

In summary, this systematic review aims to ascertain the effectiveness of customized glass fiber posts for fracture resistance in teeth that have been treated endodontically. However, study variability as well as a lack of long-term clinical data may limit the findings, emphasizing the need for more research to confirm the findings and their clinical relevance. This review’s findings will be published in scholarly journals both domestically and abroad. The PRISMA-P criteria will be followed in this review.

## Abbreviations

FRC: fiber-reinforced composite
PRISMA-P: Preferred Reporting Items for Systematic Review and Meta-Analysis Protocols
QUIN: Quality Assessment Tool for In Vitro Studies
RoB 2: Risk of Bias 2
